# An *In Silico* Approach towards the Prediction of Druglikeness Properties of Inhibitors of Plasminogen Activator Inhibitor1

**DOI:** 10.1155/2014/385418

**Published:** 2014-12-15

**Authors:** Umadevi Subramanian, Ashok Sivapunniyam, Ayyasamy Pudukadu Munusamy, Rajakumar Sundaram

**Affiliations:** ^1^Department of Marine Biotechnology, Bharathidasan University, Tiruchirappalli, Tamil Nadu 620 024, India; ^2^Department of Microbiology, Periyar University, Salem, Tamil Nadu 636 011, India

## Abstract

Diabetic retinopathy is the leading cause of blindness worldwide. It is caused by the abnormal growth of the retinal blood vessels. Plasminogen activator inhibitor1 (PAI1) is the key growth factor and the inhibition of PAI1 can reduce the angiogenesis. In this study, currently available inhibitors are taken and tested for the toxicity, binding affinity, and bioactivities of the compounds by *in silico* approach. Five toxic free inhibitors were identified, among which N-acetyl-D-glucosamine shows the significant binding affinity and two of the molecules are having the better bioactivity properties. The molecular optimization of 2-(acetylamino)-2-deoxy-A-D-glucopyranose and alpha-L-fucose can be used for the treatment of diabetic retinopathy.

## 1. Introduction

Almost half of the diabetes mellitus patients have the high risk of diabetic retinopathy. Worldwide, 17 million people are affected with proliferative diabetic retinopathy [1]. In diabetic patients, the sugar molecules accumulate in retinal blood vessels and damage them; sometimes the molecules block the vessels. Due to this, supply of oxygen and other nutrition to the retina will be reduced; this condition is called ischemia. To overcome this situation, the retina will produce new blood vessels, the process known as neovascularization. But the newly produced vessels are abnormal and fragile; they leak fluid into macula, a part of the retina which is responsible for clear central vision, and cause vision loss [2]. Presently, laser treatment is in use to treat retinopathy, but it leads to peripheral vision loss as it burns the retina. The alternative strategy is to control the expression of the growth factors which induce angiogenesis.

Plasminogen activator inhibitor1 (PAI1) is one of the growth factors responsible for neovascularization in diabetic patients. After ischemia, it is secreted from endothelial cells [3]. It is reported that inhibition of PAI1 will lead to 53% reduction in retinal angiogenesis and prevent tumor invasion and vascularization [4, 5]. In this study, an attempt was made to identify the better therapeutic inhibitor for PAI1.

## 2. Materials and Methods

### 2.1. PAI1 Structure Retrieval and Active Site Identification

The 3D structure of PAI1 was retrieved from Protein Data Bank (PDB) [6]. To identify the active site of the protein, the depth and solvent accessible surface area (SASA) were computed and based on that the probability values are assigned to each amino acid using DEPTH server [7]. The residue with high depth and SASA values are likely to form the active site.

### 2.2. Identification of Inhibitors

The inhibitor compounds (used as ligands in docking studies) so far identified against PAI1 protein were collected from various databases, namely, Human Metabolome Database (HMDB) [8], DrugBank [9], Pharmacogenomic knowledgebase (PharmaGKB) [10], and PDB.

### 2.3. Toxicity Screening

The collected ligand compounds were screened for toxicity using the online server ToxPredict (http://apps.ideaconsult.net:8080/ToxPredict). It estimates the hazard of chemical structures mainly based on Lipinski's rule and Cramer's rule. The molecules which are having the hydrogen donors ≤ 5, hydrogen bond acceptor ≤ 10, molecular mass ≤ 500 daltons, and log⁡⁡*P* ≤ 5 are likely to obey Lipinski's rule, and Cramer's rule classifies the chemical compounds into three classes based on the 33 metabolic activities. The compounds belonging to class I are of low order of toxicity, class II are more innocuous than the other two classes, and class III are of significant toxicity.

### 2.4. Docking

Docking calculations were carried out using interactive molecular graphics programs ArgusLab [11] and PatchDock [12]. Ligand was placed on a search point in the binding site which was calculated by DEPTH server; a set of diverse and energetically favorable rotations was created. In ArgusDock, exhaustive search methods for flexible ligand docking were used to calculate the binding energy. PatchDock algorithm divided the surface representation of the molecules into concave, convex, and flat patches. Then, complementary patches were matched in order to generate candidate transformations and evaluated by scoring functions. The results were visualized by Molegro Molecular Viewer (http://www.molegro.com).

### 2.5. Bioactivity Prediction

The bioactivities of the biologically significant ligands were predicted by OSIRIS Property Explorer (http://www.organicchemistry.org/prog/peo/). The calculations were originally optimized on training sets of more than 5000 compounds with measured log⁡⁡*P* values and more than 2000 compounds with measured log⁡⁡*S* values. The drug score ranges between 0 and 1.

## 3. Results and Discussion

### 3.1. PAI1 Structure Retrieval and Identification of Active Site

**Figure 1:**
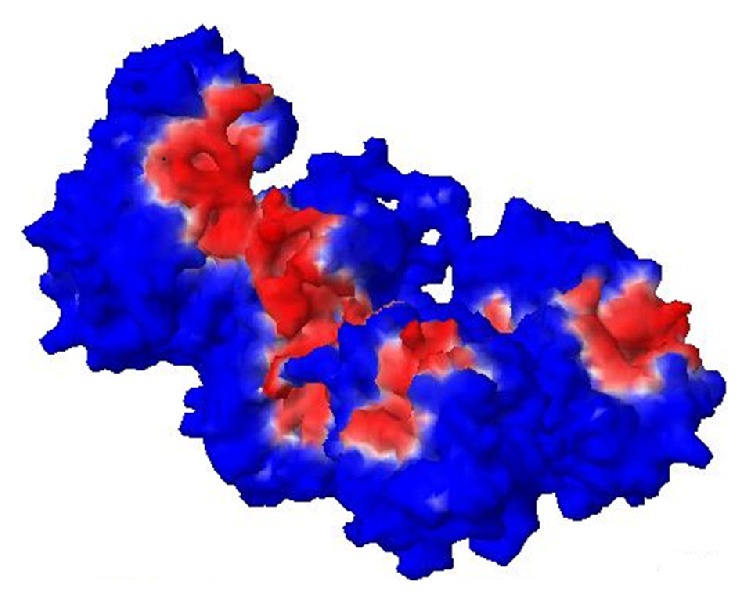
Active site region of the protein is red in colour.

**Figure 2:**
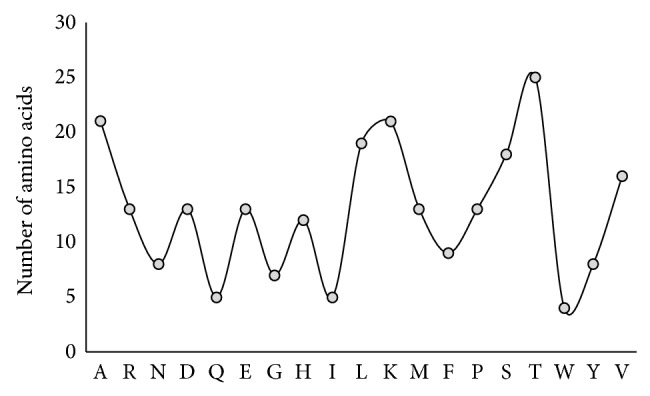
Amino acid composition of active site of PAI1.

There are 9 structures with IDs 3LW2, 3Q02, 3R4L, 1C5G, 1DB2, 1DVN, 1DVM, 1LJ5, and 1B3K which are available for PAI1 in PDB, among which the structure IB3K, which consists of 4 chains, was selected as it is in active form and is free from being bound with other molecules. The active site region was identified, represented in Figure 1, and the amino acid composition of the active site is represented in Figure 2.

### 3.2. Identified Ligand Compounds for PAI1

The inhibitors to the protein of our interest are listed in Table 1.

### 3.3. Toxicity Prediction

The toxicity of the molecules, based on Lipinski's rule and Cramer's rule whether they induce carcinogen or eye irritation, is represented in Table 2.

Based on the lower toxicity, the ligands were filtered for further studies; the molecules include 2-acetylamino-2-deoxy-A-D-glucopyranose, alpha-L-fucose, beta-D-mannose, N-acetyl-D-glucosamine, and ribose.

### 3.4. Docking Study of PAI1 with Selected Ligand Molecules

**Figure 3:**
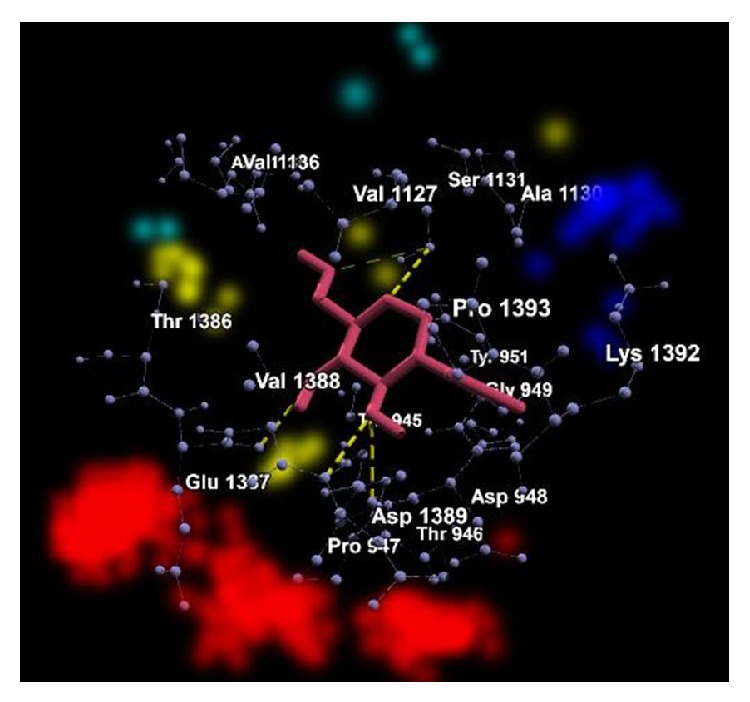
Binding of 2-acetylamino-2-deoxy-A-D-glucopyranose with PAI1.

**Figure 4:**
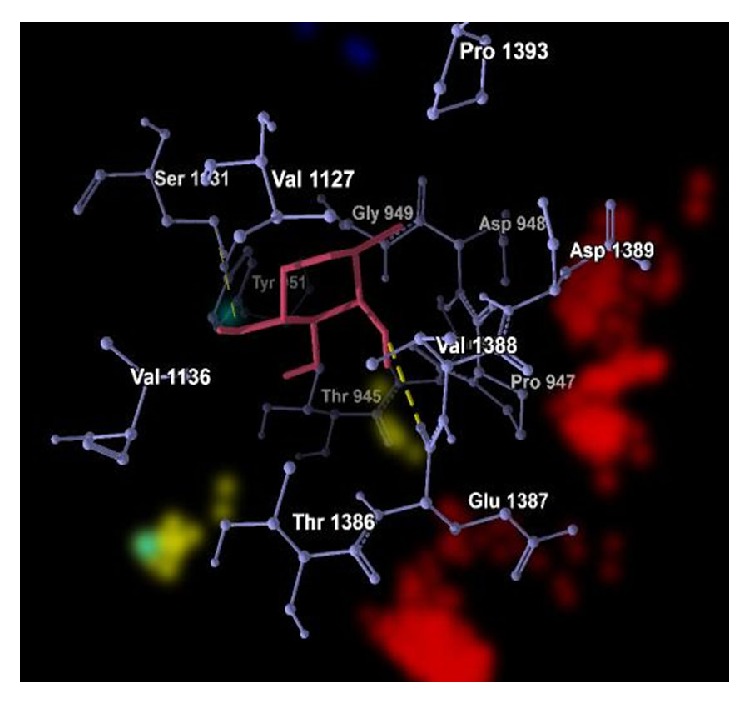
Binding of alpha-L-fucose with PAI1.

**Figure 5:**
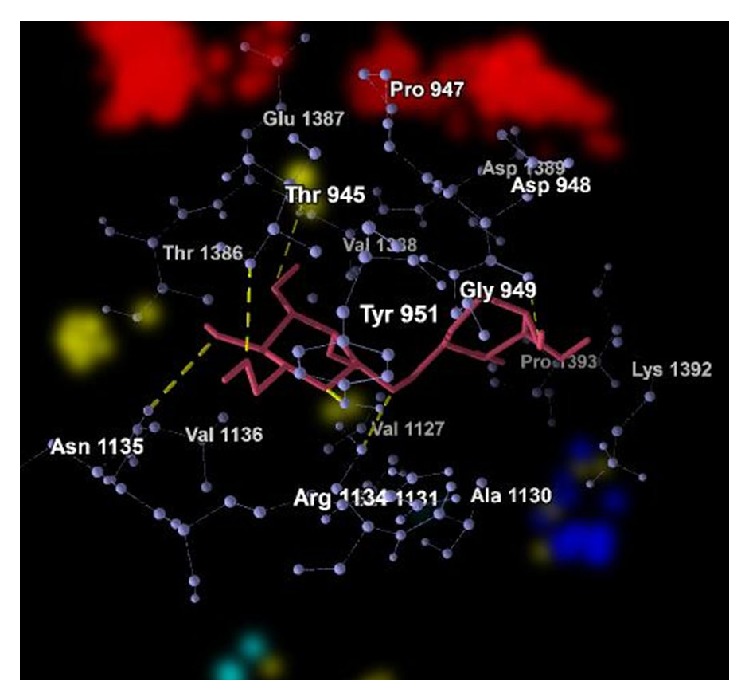
Binding of beta-D-mannose with PAI1.

**Figure 6:**
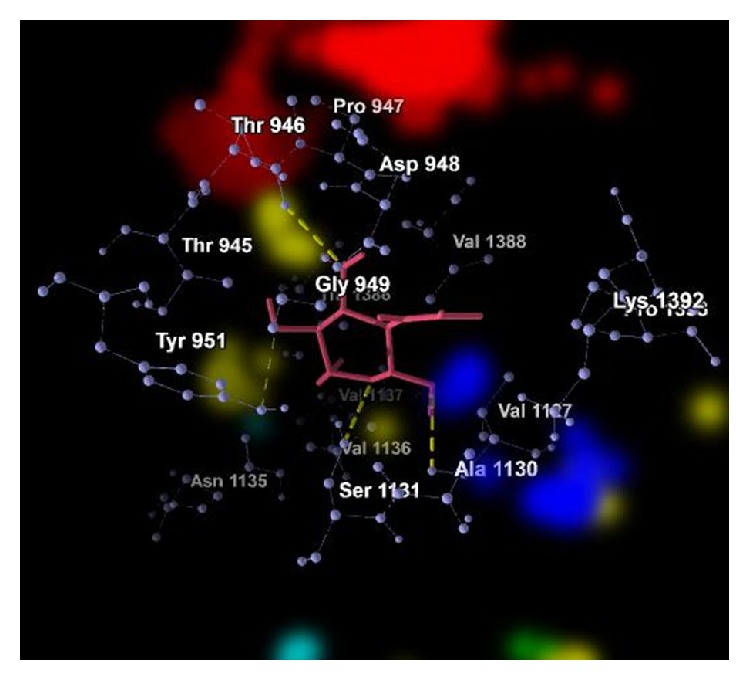
Binding of N-acetyl-D-glucosamine with PAI1.

**Figure 7:**
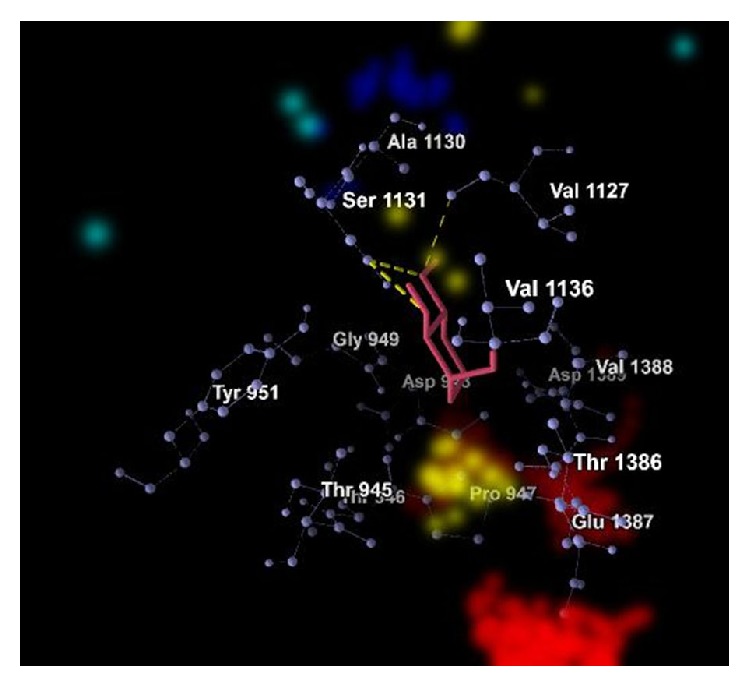
Binding of ribose with PAI1.

The graphical representations of the docking of PAI1 protein with 2-acetylamino-2-deoxy-A-D-glucopyranose, alpha-L-fucose, beta-D-mannose, N-acetyl-D-glucosamine, and ribose are in Figures 3, 4, 5, 6, and 7; yellow lines represent hydrogen bonds, pale blue dots represent hydrogen atom acceptors, yellow dots represent hydrogen atom donors, red dots represent positive ions, and dark blue dots represent negative ions. The corresponding binding energy values are presented in Tables 3, 4, 5, 6, and 7 and the overall energy of each inhibitor binding with PAI1 is in Table 8.

### 3.5. Bioactivity Properties

The bioactivity of the ligands which are toxic free and having biologically significant binding affinity is represented in Table 9.

Among the presently identified inhibitors against PAI1, only five of them, namely, 1,2-ethanediol, beta-D-mannose, alpha-L-fucose, N-acetyl-D-glucosamine, and 2-(acetylamino)-2-deoxy-A-D-glucopyranose, come under class I of Cramer's rule. As 1,2-ethanediol causes the irritation in eye, it is excluded from the study. The remaining ligand compounds are used for further study.

The docking results showed that N-acetyl-D-glucosamine is highly biologically significant followed by 2-(acetylamino)-2-deoxy-A-D-glucopyranose, beta-D-mannose, alpha-L-fucose, and ribose in decreasing order. The above five molecules are toxic free and can bind with PAI1, but the bioactivities of the compounds revealed that 2-(acetylamino)-2-deoxy-A-D-glucopyranose and alpha-L-fucose are having the property of druglikeness at moderate level; the rest cannot be used for the purpose of drug.

## 4. Conclusion

For the known inhibitors of PAI1, toxicity, binding affinity, and bioactivity were predicted computationally. There were five molecules identified; moreover they have the feasible binding affinity with PAI1 as well. As the molecular weight of N-acetyl-D-glucosamine and beta-D-mannose is higher and *c*Log *P* value is higher for ribose, there is a least priority to these compounds to be used as drug. The molecules 2-(acetylamino)-2-deoxy-A-D-glucopyranose and alpha-L-fucose were identified as better therapeutic inhibitors to PAI1 than other molecules used in this study. Due to the toxic free nature and significant binding energy, this study can be extended at clinical level. For the efficient and quick treatment level, they should be structurally optimized as the drug score of the identified two molecules was moderate.

## Figures and Tables

**Table 1:** Ligand molecules of PAI1 protein.

Database	Inhibitor molecules
HMDB	Atorvastatin, dimethyl sulfoxide, and simvastatin.
DrugBank	Troglitazone.
PharmaGKB	Antidepressants (including amitriptyline hydrochloride, amoxapine, clomipramine hydrochloride, and desipramine hydrochloride), citalopram, and fluoxetine.
PDB	2,5-Dihydroxy-3-undecyclohexa-2,5-diene-1,4,-dione; 1,2-ethanediol; beta-D-mannose; alpha-L-fucose; N-acetyl-D-glucosamine; 2-acetylamino-2-deoxy-A-D-glucopyranose; ribose; acetic acid.

**Table 2:** Toxicity prediction of the inhibitors of PAI1.

Ligand molecule	Obey Lipinski's rule?	Cramer's rule	Carcinogen	Eye irritation
Troglitazone	Yes	Class III	No	No
Dimethyl sulfoxide	Yes	Class III	No	No
Atorvastatin	No	Class III	No	No
Simvastatin	Yes	Class III	No	No
Citalopram	Yes	Class III	No	No
Fluoxetine	Yes	Class III	No	No
2,5,Dihydroxy-3-undecyciohexa-2,5-diene-1,4,dione	No	Class III	No	No
1,2-Ethanediol	Yes	Class I	No	Yes
Beta-D-mannose	Yes	Class I	No	No
Alpha-L-fucose	Yes	Class I	No	No
N-Acetyl-D-glucosamine	Yes	Class I	No	No
2-(Acetylamino)-2-deoxy-A-D- glucopyranose	Yes	Class I	No	No
Ribose	Yes	Class I	No	No
Acetic acid	Yes	Class III	No	No
Amitriptyline hydroxychloride	No	Class III	No	No
Amoxapine	Yes	Class III	No	No
Clomipramine hydrochloride	Yes	Class III	No	No
Desipramine hydrochloride	Yes	Class III	No	No

**Table 3:** Binding energy of 2-acetylamino-2-deoxy-A-D-glucopyranose with PAI1.

Bond	Energy		Residues involved
Hydrogen	−2.5 (kcal/mol)	2.64532 Å (length)	Glu 1387
Hydrogen	−2.5 (kcal/mol)	2.99992 Å (length)	Val 1388
Hydrogen	−2.5 (kcal/mol)	2.69638 Å (length)	Pro 947
Hydrogen	−0.835562 (kcal/mol)	3.43289 Å (length)	Ser 1131
Hydrogen	−2.5 (kcal/mol)	2.72334 Å (length)	Ser 1131

Hydrogen (nondirectional)	−13.551 (kcal/mol)		—

Steric	−71.96 (kcal/mol) (by PLP) −24.70 (kcal/mol) (by LJ16-6)		Asp 948, Gly 949, Thr 945, Thr 946, Thr 951, Ala 1130, Asn 1135, Asp 1389, Lys 1392, Pro 1393, Thr 1386, Val 1127, Val 1136

**Table 4:** Binding energy of alpha-L-fucose with PAI1.

Bond	Energy		Residues involved
Hydrogen	−2.50 (kcal/mol)	2.84262 Å (length)	Val 1388
Hydrogen	−0.91 (kcal/mol)	3.41776 Å (length)	Ser 1131

Hydrogen (nondirectional)	−4.04 (kcal/mol)		—

Steric	−52.65 (kcal/mol) (by PLP) −17.65 (kcal/mol) (by LJ16-6)		Asp 948, Gly 949, Pro 947, Thr 945, Thr 946, Tyr 951, Ala 1130, Asp 1389, Glu 1387, Pro 1393, Thr 1386, Val 1127, Val 1136

**Table 5:** Binding energy of beta-D-mannose with PAI1.

Bond	Energy		Residues involved
Hydrogen	−1.34 (kcal/mol)	3.05 Å (length)	Glu 1387
Hydrogen	−0.23 (kcal/mol)	3.51 Å (length)	Glu 1387
Hydrogen	−2.45 (kcal/mol)	3.11 Å (length)	Val 1136
Hydrogen	−1.78 (kcal/mol)	3.24 Å (length)	Thr 945
Hydrogen	−2.50 (kcal/mol)	2.86 Å (length)	Tyr 951

Hydrogen (nondirectional)	−15.91 (kcal/mol)		—

Steric	−35.10 (kcal/mol) (by PLP) −445.37 (kcal/mol) (by LJ16-6)		Asp 948, Gly 949, His 950, Thr 946, Ala 1130, Arg 1134, Asn 1135, Asp 1389, Gln 1126, Lys 1392, Pro 1393, Thr 1386, Val 1127, Val 1388

**Table 6:** Binding energy of N-acetyl-D-glucosamine with PAI1.

Bond	Energy		Residues involved
Hydrogen	−2.50 (kcal/mol)	2.85 Å (length)	Val 1127
Hydrogen	−0.21 (kcal/mol)	3.55 Å (length)	Ser 1131
Hydrogen	−2.50 (kcal/mol)	3.02 Å (length)	Ser 1131
Hydrogen	−0.90 (kcal/mol)	3.42 Å (length)	Tyr 951
Hydrogen	−2.50 (kcal/mol)	2.84 Å (length)	Pro 947

Hydrogen (nondirectional)	−14.54 (kcal/mol)		—

Steric	−44.44 (kcal/mol) (by PLP) 13.40 (kcal/mol) (by LJ16-6)		Asp 948, Gly 949, His 950, Thr 945, Thr 946, Ala 1130, Asn 1135, Asp 1389, Gln 1126, Glu 1387, Lys 1392, Pro 1393, Thr 1386, Val 1136, Val 1388

**Table 7:** Binding energy of ribose with PAI1.

Bond	Energy		Residues involved
Hydrogen	−1.02 (kcal/mol)	3.39 Å (length)	Val 1127
Hydrogen	−2.30 (kcal/mol)	2.57 Å (length)	Ser 1131
Hydrogen	−2.49 (kcal/mol)	2.59 Å (length)	Ser 1131

Hydrogen (nondirectional)	−5.82 (kcal/mol)		—

Steric	−46.01 (kcal/mol) (by PLP) −17.31 (kcal/mol) (by LJ16-6)		Asp 948, Gly 949, Pro 947, Thr 945, Thr 946, Tyr 951, Ala 1130, Asp 1389, Glu 1387, Thr 1386, Val 1136, Val 1388

**Table 8:** The overall binding affinity of ligands with PAI1.

Ligand molecule	Binding energy (kcal/mol)	Area covered (Å)
N-Acetyl-D-glucosamine	−7.83	386.10
2-Acetylamino-2-deoxy-A-D-glucopyranose	−6.03	402.30
Beta-D-mannose	−6.00	508.40
Alpha-L-fucose	−5.43	283.50
Ribose	−5.13	251.00

**Table 9:** Bioactivity of the selected ligands of PAI1.

Ligand	clog⁡P	Solubility	Molecular weight	Druglikeness	Drug score
2-Acetylamino-2-deoxy-A-D-glucopyranose	−1.63	−0.15	191.01	−0.50	0.67
Alpha-L-fucose	−1.63	−0.15	101.00	−0.56	0.67
Beta-D-mannose	−3.5	0.42	312.00	−5.08	0.38
N-Acetyl-D-glucosamine	−2.3	−0.02	221.00	−3.05	0.5
Ribose	−1.3	−0.06	134.00	−5.68	0.4
